# Helmet Therapy for Deformational Plagiocephaly: Clinical Outcomes and Considerations

**DOI:** 10.3390/children12121597

**Published:** 2025-11-24

**Authors:** Sum-Yuet Ching, Oscar Chun-Yiu Wong, Wai-Wang Chau, Alec Lik-Hang Hung, Tsz-Ping Lam, Adam Yiu-Chung Lau

**Affiliations:** 1 Prosthetic and Orthotics Department, Prince of Wales Hospital, Hong Kong SAR, China; csy447a@ha.org.hk (S.-Y.C.);; 2 Department of Orthopaedics and Traumatology, The Chinese University of Hong Kong, Hong Kong SAR, China; jackchau@cuhk.edu.hk (W.-W.C.);; 3 Department of Orthopaedics and Traumatology, Prince of Wales Hospital, Hong Kong SAR, China

**Keywords:** helmet therapy, deformational plagiocephaly, cranial remolding orthosis, positional plagiocephaly

## Abstract

**Highlights:**

**What are the main findings?**

- Helmet therapy significantly reduces head deformity of infants with deformational plagiocephaly.
- Wearing the helmet for at least 6 h per day during sleep improves head shape.
- Low helmet compliance (0–5 h/day) results in no improvement or deterioration, whereas all patients with high compliance (≥18 h/day) demonstrate improvement.

**What is the implications of the main findings?**

- Early initiation of helmet therapy and good compliance, particularly nighttime use, is strongly encouraged to enhance the effectiveness of helmet therapy.

**Abstract:**

**Background/Objectives**: Deformational plagiocephaly (DP) is defined as an asymmetrical flattening of infants’ occipital skull. Helmet therapy is one of the treatments reported that can reduce skull deformity by guiding the growing direction of the head. However, its effectiveness remains insufficiently validated in the literature due to variability in treatment protocols and regimes. This study aims to evaluate the clinical outcomes of helmet therapy in infants with deformational plagiocephaly. **Methods**: This single-center retrospective study was conducted at a tertiary university hospital and included 30 consecutive infants (mean age 7.83 ± 2.51 months) who attended a pediatric orthopedic outpatient clinic between 2022 and 2025. Infants without craniosynostosis and with cranial vault asymmetry index (CVAI) ≥ 5% were prescribed a course of helmet therapy (mean duration 3.77 ± 2.37 months). The primary outcome was the change in CVAI. **Results**: The mean CVAI (%) significantly decreased from 7.57 ± 2.45 to 6.10 ± 2.63 (*p* = 0.002). The effect of helmet therapy was dose-dependent, with greater improvement observed in infants wearing the helmet for at least 6 h per day. Poor compliance and predominantly daytime helmet use were associated with less improvement. Increased sweating and mild skin redness were the most common reported adverse effects, but the skin redness can be relieved by proper donning of the helmet. **Conclusions**: Helmet therapy is effective for infants with plagiocephaly when initiated early and with high compliance of helmet use. Greater improvements were observed in infants using the helmet during sleeping. Further multi-center studies with a larger sample size and longer follow-up are recommended.

## 1. Introduction

Deformational plagiocephaly (DP), also referred to as non-synostotic plagiocephaly or positional plagiocephaly, is a condition defined as the asymmetrical flattening of infants’ occipital skull, specifically those between 6 weeks and 1 year of age [1]. Different from unilateral coronal synostosis (UCS) and lambdoid synostosis, which also cause oblique deformities of the head by premature closure of coronal suture [2], DP is associated with a constant external pressure applied to the growing infant’s head [3]. Skull shape naturally changes within the first 2 years of life, while 85% of cranial growth occurs in the first year [4,5]. Asymmetric baby head shapes increased after the “Back to sleep” campaign in 1992 which recommended supine sleeping positions for healthier infants [6,7]. Infants resting in a static supine position on a rigid, flat surface limits the cranial growth and thus flattening becomes more obvious [8]. Especially for individuals with congenital muscular torticollis (CMT), the muscular imbalance is visible as asymmetric head rotation with better movement to one side, and infants will prefer to rest on the flattened side [9]. Not only will this deformed skull condition lead to facial asymmetry, but the risk of developmental delay will also be increased [10,11].

Early initiation of conservative treatment such as repositioning, physical therapy, and manual therapy is effective in reducing mild head deformity [12]. Educating caregivers to give motor, sensory, and repositioning stimulation of the non-preferred sides as well as prone positions is also suggested as a primary treatment for any degree of DP [13]. Exercises that stimulate motor development and reduce positional preference can prevent deterioration of deformity before the age of 12 months [14]. Molding and decompression techniques used in manual therapy can further shorten the treatment duration for patients with severe DP and thereby reduce the risk of DP-related complications [15].

The use of a customized cranial remolding orthosis (helmet) is suggested for moderate to severe cases [16,17]. Since the cranium expands to areas with less resistance, the custom-designed helmet can guide the growth of the skull by relieving the flattened area [18].

Since the 1980s, studies have been performed to prove the effectiveness of helmets in head shape improvement. Over the years, more evidence supports that helmet therapy significantly raises the correction rate in infants with moderate or severe DP [16,19,20,21]. Early initiation of the therapy and high compliance during treatment can further improve the outcome [18,22,23,24,25,26].

In Japan, helmet therapy has been evaluated to be safe and effective in treating DP [26]. Statistically significance improvements were shown in both 2D and 3D metrics [27]. A study in Korea has also shown that helmet therapy is effective for infants with DP, especially for those who start treatment before 9 months and use the helmet at least 15 h per day [25].

However, the efficacy of helmet therapy has still not been fully validated. In a systematic review conducted in 2024, it was not feasible to compare across studies due to the lack of a standardized treatment plan [28]. Most studies did not include all treatment details in their study designs. The diagnosis of severity of DP, successful outcome measures, and the definition of a successful treatment were different among studies, which makes it difficult to conclude a solid recommendation for helmet therapy [29]. Also, there was only one randomized control trial that yielded level 1 evidence, while most articles reported retrospective case series. The evidence level regarding the effects of helmet therapy was difficult to conclude due to the lack of high-quality studies as well as a universal standard [13].

This study aims to review the results from the current helmet therapy treatment plan for local patients with DP in Hong Kong. Factors affecting success of treatment, including initial severity of the deformity, initiation age, compliance of helmet use, and duration of treatment, will be analyzed [30].

## 2. Materials and Methods

This is a single-center retrospective case review study conducted in a tertiary university hospital. Ethics approval had been obtained from the local ethics committee (Ethics approval number: CREC Ref. No. 2024.385).

### 2.1. Patients and Patient Recruitment

**Figure 1:**
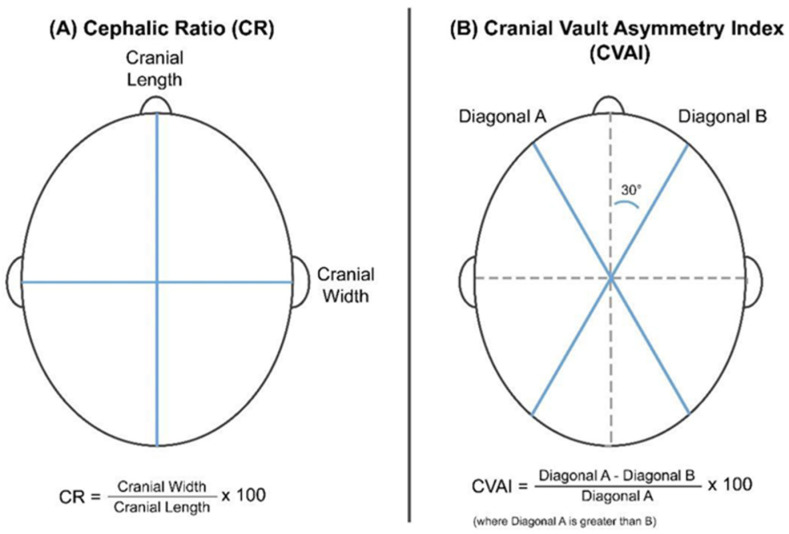
Cephalic ratio (CR) and CVAI calculation [31].

**Figure 2:**
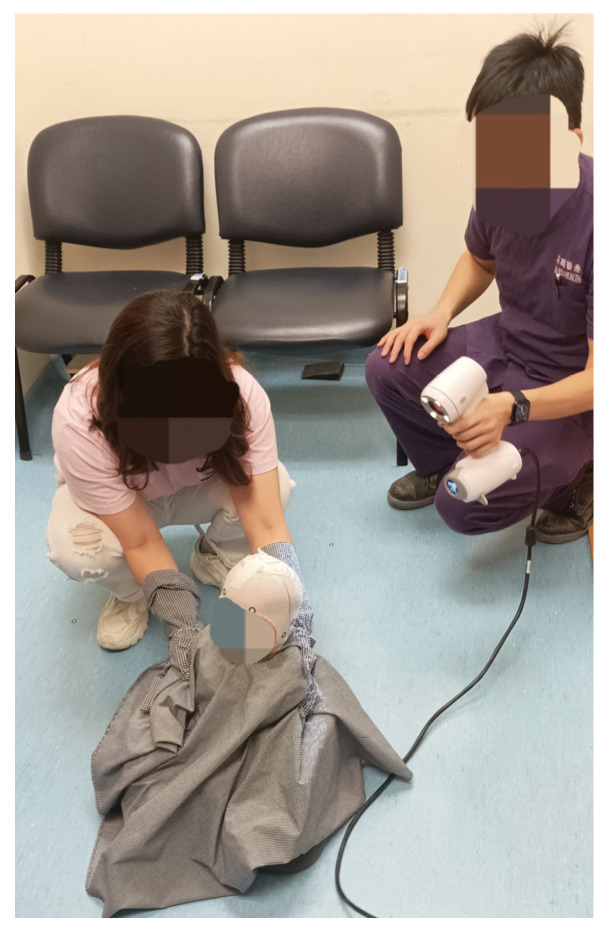
Scanning set-up.

Patients were recruited in the Pediatric Orthopedics Unit, Department of Orthopedics and Traumatology, from a local tertiary hospital. Inclusion criteria were as follows: (1) a clinical diagnosis of positional plagiocephaly with cranial vault asymmetry index (CVAI) ≥ 5% (Figure 1); (2) age between 3 and 12 months in the first fitting session; (3) complete clinical records throughout the treatment. All head scans and helmet fabrication were managed by the Prosthetics and Orthotics Department in the same hospital. Patients were excluded from this study when (1) the deformity of the head was caused by early closure of the skull, i.e., synostosis, or (2) their head was deformed in the AP (brachycephaly) or ML (scaphocephaly) direction only.

Details of treatment outcomes were stored electronically and hard copies from patients who received helmet therapy consecutively from the year 2022 to 2025 were reviewed retrospectively. The change in CVAI before and after treatment was analyzed to assess the efficacy of helmets.

### 2.2. Treatment

All patients had anthropometric measurements collected by a 3D scanner (Model name: M4D Scan, Manufacturer: Rodin4D, City: Pessac, Country: France). During scanning, patients were secured in an infant car seat and supported by their parents/legal guardians or experienced therapists to minimize imaging noise (Figure 2). The measurements, such as diagonal difference and CVAI, were calculated in the modeling software Rodin Neo (Rodin4D version 10.2). The scanned file was then refined within the software, including smoothing the surface and relieving the flattened part to guide the skull’s growth. A maximum of 1 cm expansion was given to prevent loosening of the helmet. The area of the bilateral cheek and posterior neck distal to the occipital was slightly reduced to improve the suspension of the helmets.

**Figure 3:**
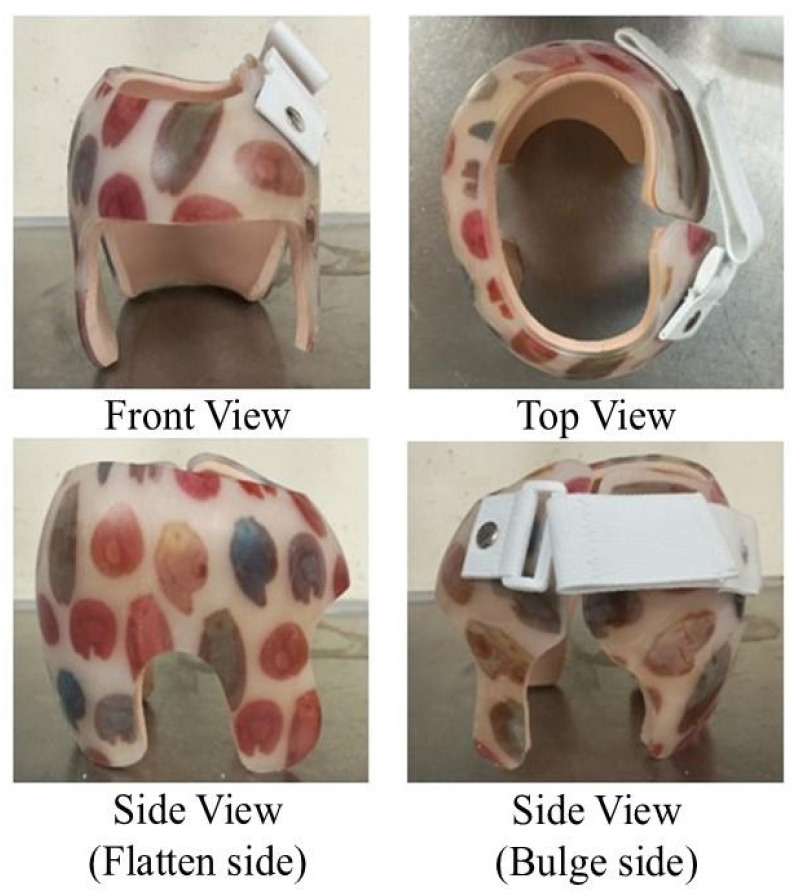
Helmet design with plastazote inner padding.

The helmet was fabricated by thermoforming on the modified positive cast by a single artisan. The helmet consists of two parts: inner padding for comfort and an outer shell for rigid support. Either plastazote or Comfortex Air (Ottobock) was used as the inner padding, while polypropylene was used as the outer shell (Figure 3). The upper trimline of the helmets was opened for better ventilation. The opening of the helmet and a strap were set on the opposite side of the flattened posterior head for better circumferential force for suspension. All helmets used the same material and followed the same rules for design except for the inner padding, depending on any allergies to the material. To ensure the fitness of the helmet, the fitting time was controlled within 1 month after the first appointment.

After fitting the helmet, parents/legal guardians were instructed to increase the donning time of the helmet gradually up to 23 h per day after a break-in period of 1–2 weeks. Cleanliness and good hygiene of the helmet were important to keep up with to minimize skin irritations. Regular ventilation, such as checking the skin condition and cleaning the infant’s head, was suggested to prevent pressure sores on the non-flattened side. The helmet was recommended to be used during sleep to prevent pressure acting on the flattened skull. If there were no complications, the helmet was used until the patients reached 18 months old. They were advised to stop using the helmet immediately when any red or discolored skin patches were observed, especially at the beginning of the treatment. A follow-up session was arranged to adjust the helmet, and helmet use was resumed once skin problems had been resolved.

Regular follow-up sessions were arranged every 1–2 months after fitting the helmet to check helmet fitness, change in CVAI, and compliance of helmet use. In each follow-up session, scanning was performed to trace the head shape change. CVAI (%), compliance of helmet use (hours/day), and wearing pattern (daytime, nighttime, full-time) were collected. Comments were also recorded from parents/legal guardians regarding the quality of life when using the helmet and any complications throughout the treatment. The follow-up sessions were arranged until the age of 18 months [22]. Even if patients stopped using the helmet earlier, any change in head shape was traced until 18 months.

### 2.3. Data Collected

Demographic characteristics were collected, such as age at recruitment and sex. CVAI was used to define the severity of deformity and the improvements of deformity. CVAI refers to the percentage of diagonal difference at the eyebrow level (Figure 1). Deformity level was divided into 3 levels in terms of CVAI: <6% was mild, 6–10% was moderate, and >10% was severe. The improvement of head shape was defined as the difference in CVAI before and after treatment. The compliance of helmet use and wearing pattern were recorded through their guardians’ self-report. Compliance was classified as excellent (≥18 h/day), good (12–17 h/day), fair (6–11 h/day), or poor (<6 h/day). The classification of wearing patterns was determined by whether the helmet was used during sleeping or in daytime activities. If the helmet was used during sleeping only, it would be classified as nighttime use.

### 2.4. Statistical Analysis

The primary outcome was the improvement in CVAI, which was evaluated using a paired T-test. The change in CVAI was calculated by subtracting the final CVAI from the initial CVAI before wean off. Initial CVAI (categorized) and final CVAI before wean off (categorized) were cross-tabulated to show the changes in status before and after helmet therapy. The relationship between helmet compliance and the change in CVAI after helmet therapy was assessed. Linear regression models on the change in CVAI were carried out for (1) age at recruitment, (2) sex, (3) initial deformity level, (4) helmet compliance, and (5) wearing pattern. Stepwise linear regression followed when any factor was found to be statistically significant. Dummy variables were created for categorical variables. All statistical analyses were carried out using IBM SPSS version 29 (Armonk, NY, USA: IBM Corp). Statistical significance was set at *p* < 0.05.

## 3. Results

Thirty patients, aged 4 to 12 months old at recruitment, were enrolled. Of these, twenty-one were male. Congenital muscular torticollis (CMT) was the most associated comorbidity (Table 1). In addition to helmet therapy, twenty-two patients also underwent physiotherapy during the treatment process. There were two pairs of twins, and two infants also presented with brachycephaly.

One-third of the patients began helmet use before 7 months of age (Table 1). As the majority of patients in our clinic had tried non-orthotic treatments before helmet therapy, most of their first orthotic appointments occurred after 6 months of age. This caused delayed initiation of treatment. In view of this situation, half of the patients started helmet therapy at 7–9 months of age. The mean age at the final session was 12.03 months, and five patients were followed up continuously until 18 months of age.

After helmet therapy, improvements were found in 56.7% (*n* = 17) of patients and 23.3% (*n* = 7) remained unchanged, with 14 patients (46.7%) having a CVAI of ≤5% at the final session. The mean CVAI (%) was significantly reduced from 7.57 ± 2.45 to 6.10 ± 2.63 (mean ± SD, *p* = 0.002, Table 2). The mean change in CVAI was 1.47 ± 2.42 (median = 1.50; range = −3 to 7). Details of the change in CVAI are presented in Table 3.

When examining changes in CVAI categories before and after helmet therapy, all mild cases (*n* = 4) at baseline remained mild after helmet therapy (Table 4). Among the 23 patients with an initial moderate deformity, 10 (43.5%) improved to mild, 12 (52.2%) remained at moderate, and 1 (4.3%) deteriorated to severe. Of the three patients with severe initial CVAI, two (66.7%) improved to moderate and one (33.3%) remained at severe.

A statistically significant association was found between helmet compliance and changes in CVAI (*p* = 0.049) (Table 5). Among the 11 patients with helmet compliance between 0 and 5 h/day, 9 (81.8%) showed no change or deterioration in CVAI. All patients with an average of 18 or more hours/day of compliance demonstrated improvement in CVAI.

Linear regression modeling showed that compliance of helmet use for 1) 6–11 h/day (B(95% CI) = 2.557 (0.492, 4.622); SE = 1.005; *p* = 0.017), 2) 12–17 h/day (B(95% CI) = 2.515 (0.518, 4.512); SE = 0.972; *p* = 0.016), and 3) 18+ h/day (B(95% CI) = 3.182 (0.334, 6.598); SE = 1.102; *p* = 0.027) had significantly positive impacts on the change in CVAI (Table 6). Other independent variables did not show statistical significance. Stepwise linear regression modeling further indicated that compliance of 12–17 h/day (B(95% CI) = 2.655 (0.101, 5.209); *p* = 0.042) and 18+ h/day (B (95% CI) = 3.194 (0.046, 7.133); *p* = 0.048) had significantly positive impacts on CVAI changes, regardless of age at recruitment, sex, and initial deformity (Table 7). Compliance of helmet use at 6–11 h/day (B (95% CI) = 2.416 (0.216, 4.615); *p* = 0.033) also showed a significant positive impact, independent of initial age and sex.

**Figure 4:**
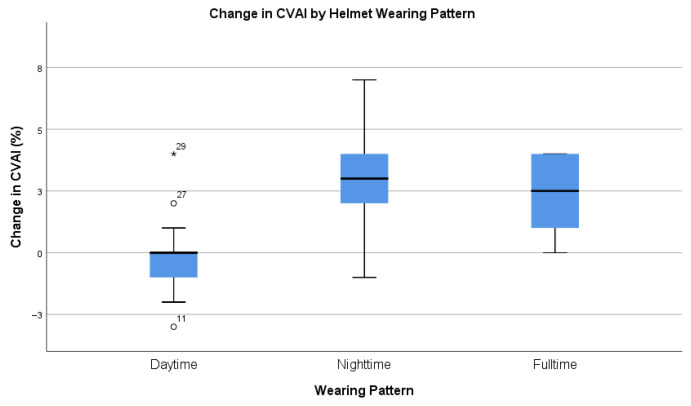
Change in CVAI by helmet wearing pattern.

When patients were grouped according to their helmet wearing patterns (Figure 4, Table 8), the average change in CVAI was greater in those who wore the helmet while sleeping or fulltime compared to those who wore it during the daytime only (one-way ANOVA, F(2,27) = 7.769, *p* = 0.002).

**Figure 5:**
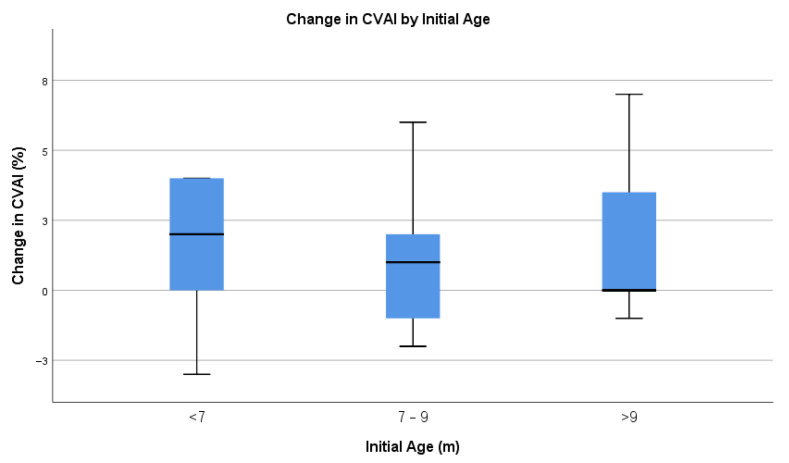
Change in CVAI by initial age.

When dividing infants by their initial age (Figure 5, Table 9), it was not statistically significant (one-way ANOVA, F(2,27) = 1.189, *p* = 0.829). The average change in CVAI was similar among groups and the ranges in each group were large.

Among all recruited subjects, there were two pairs of twins, and in each pair, one infant also had brachycephaly. The compliance and wearing pattern were 0–6 h/day in daytime for pair 1, while pair 2 wore the helmet for 6–12 h/day at night. The two patients who also had brachycephaly showed improvement mainly in the anteroposterior–mediolateral (AP-ML) difference, while diagonal deformity remained similar (Figure 1, Table 10).

All parents/legal guardians reported skin redness or mild irritation found in the first follow-up session. Most of the cases were resolved after education on proper use of the helmets (e.g., hygiene and how to position the helmet). One mother reported skin breakdown after the first fitting of the helmet. She was informed to stop using the helmet immediately and the skin recovered after 1 week. Since the patient’s skin could not tolerate the pressure, the helmet was adjusted immediately. No further complications were reported after the adjustment.

## 4. Discussion

The statistically significant improvement in CVAI shows the effectiveness of helmets in treating patients with DP (Table 2). The result coincided with similar studies conducted in other countries. For example, a study conducted by a team led by Takamatsu has already demonstrated the effects of helmet therapy in Argenta classification, and CA and CVAI were statistically significant [26]. Among 366 Japanese infants with DP, the average CVAI reduced from 12.9% to 5.4% after receiving an average of 21.2 weeks of treatment. Kluba, Kraut, Calgeer, Reinert, and Krimmel [17] also performed a prospective longitudinal study to evaluate the effectiveness of helmet therapy in improving CVAI. The average CVAI of 62 infants with severe DP was improved from 13.3% to 4.1%.

### 4.1. Factors Affecting Treatment Efficacy

Improvement in head shape was observed when helmet compliance was ≥6 h (Table 5 and Table 6). Wearing the helmet for at least 6 h per day resulted in a significant reduction in CVAI. Patients with low compliance of helmet use (0–5 h per day) showed no improvements or even deterioration, whereas improvements were found in all patients with high compliance (18+ h per day). Change in CVAI is positively associated with compliance of helmet use: the higher the compliance, the higher the change in CVAI. A previous study demonstrated that higher compliance with helmets was statistically significant in increasing the correction rate of head shape [32]. From the findings of this study, a minimum helmet compliance of 6 h per day is recommended to reflect a better treatment outcome.

The improvement in deformity was more significant in those who used the helmet at nighttime compared to those who used it for longer times in the daytime. As shown in Figure 4 and Table 8, the mean change in CVAI in the daytime group is less than the other two groups. One important point we need to look at is that there are three outliers in the daytime group. The causes of the outliers are multi-factorial: for case 11, the self-reported helmet compliance was 2 h per day (low compliance), while cases 27 and 29 started therapy before the age of 6 months (early initiation). Nonetheless, this is the first study to propose the effect of nighttime bracing for DP patients. Further study is warranted to further consolidate this finding. At this moment, we encourage caregivers to pay particular attention to the use of helmets at nighttime.

The initial severity before treatment should also be considered. Patients with moderate to severe deformity showed greater improvements after helmet therapy (Table 4). Improvements in CVAI were observed in patients with moderate to high compliance, regardless of age at recruitment, sex, and initial deformity. However, it was not statistically significant in groups with mild initial deformity and low compliance. This finding is consistent with a study from Korea [25], which reported that helmet therapy is more suitable for infants with moderate to severe deformities.

The starting age of treatment is a crucial factor influencing outcomes. Although the result was not statistically significant (Table 9), likely due to the limited sample size and high variability within groups, initiating helmet therapy before 7 months of age [18] shows the highest mean change in CVAI, except for one isolated case that deteriorated by 3%. Beyond the commonly cited reason that infants’ skulls become less malleable as they grow, this study identified a new observation: as the patients grow older, caregivers frequently reported that infants would pull off the helmet themselves. This behavior was more common in those who began helmet therapy after 10 months of age. Such resistance and incorporation significantly reduced helmet compliance, thereby affecting treatment effectiveness. This may be another reason why early initiation of helmet therapy can enhance acceptance and adaptation to the helmet.

The progress of patients with both DP and brachycephaly should be highlighted. For patients with complex head deformities, their head shape showed improvement in the AP–ML difference but kept similar CVAIs. This might be due to the design of the helmet and the fact that the relief area was more than a quarter of the head circumference. Hence, the head grew towards the AP direction instead of diagonally. Longer observation is needed to investigate the correction progress of patients with complex types of head deformities.

“Proxy” help from grandparents is gaining popularity around the world, particularly during the economic downturn in recent decades [33]. Both parents opt to work to earn a sustainable living [34]. Moreover, living with grandparents is a growing trend for families to save housing expenses. As a result, grandparents become the main caregivers to babies. During this study, one mother of a recruited infant reported that, as the grandparents were the main caregivers, it was very difficult to maintain proper helmet compliance, as the parents were often out of sight. Moreover, the strong thought of acting against the use of helmet on the DP baby from grandparents made the helmet compliance even worse. Through the experience gained from this study, we strongly recommend healthcare providers to discuss with the parents and any “proxy” caregivers, such as grandparents, legal guardians, or other people who play a chief role in the care of the DP baby, explicitly about the objectives and importance of using the helmet in a proper way at this age.

Two pairs of twins were recruited in this study. Referring to Table 10, patient 1a and patient 1b were a pair of twins with different helmet therapy starting ages. While both patients visited our clinic on the same day, patient 1a’s CVAI was <5% at that moment. Since patient 1b’s brachycephaly was severe, the caregiver agreed to commence the helmet therapy while the decision was “under observation” for patient 1a. As time went on, patient 1a’s CVAI increased to 6% at the next visit. Patients eventually agreed to start the therapy for patient 1a. This example showed that healthcare professionals should prompt the caregivers that delaying treatment could possibly lead to an increase in deformity, leaving the best time window closed.

### 4.2. Safety of Helmet Therapy

Increased sweating and mild skin irritations were common adverse effects after using the helmet. This observation coincides with the study in Japan [35] and occurred even more commonly due to the hot subtropical weather in the subtropical region. All caregivers reported they had to turn on the air conditioner every day, even during winter, in order to keep a comfortable ambient environment for the infants with the helmets. Although it might have caused an inconvenience in their daily life, they were able to adapt to the new living practices within 1 month. Skin redness and skin irritations were resolved after the proper donning and removal of the helmet and after increasing the wearing time of the helmet gradually. Only one case reported skin breakdown after the first fitting of the helmet and skin problems were resolved after adjustment of the helmet. No change in behavior was observed for all patients. Overall, helmet therapy is safe for infants with DP. Parents or guardians should be clearly informed about the proper usage of the helmet and the possible skin problems, especially at the early stage of treatment.

### 4.3. Limitations of This Study

This study has several limitations. First, the small sample size increases the risk of both type I and II errors in this study. Second, the absence of a comparator group (e.g., infants with physiotherapy training only) introduces performance bias and limits the strength of the conclusion. Third, reliance on subjective compliance data from parents may lead to measurement bias. A similar study has already demonstrated the difference between subjective and objective monitoring of helmet compliance [36], which reduces the accuracy of the reported dose–response relationship. Fourth, the majority of this cohort received their final head shape assessment at 12 months. After the helmet was weaned off, most patients did not attend the scheduled follow-up session, and only five patients underwent a CVAI assessment at 18 months. Potential changes in head shapes after discontinuing helmet use could not be ruled out. Longitudinal monitoring of the head shape up to 18 months of age for this cohort, regardless of the usage of helmets, is highly recommended.

### 4.4. Further Study

A multi-center, large-scale longitudinal study or randomized trial is recommended to compare the treatment outcomes. A robust long-term follow-up period is also recommended to monitor the changes in head shape as the infants grow. Incorporating pressure or temperature sensors inside the helmet can improve the reliability of compliance data collection [30].

## 5. Conclusions

This study demonstrated that head shape (CVAI) can be improved through helmet therapy in infants with deformational plagiocephaly. Early initiation of helmet therapy, high compliance (≥18 h/day) during treatment, and greater initial severity were associated with more substantial percentage improvements post-treatment. Wearing the helmet for at least 6 h during sleep can further enhance treatment effectiveness. A long-term study with a larger sample size and objective compliance monitoring is recommended to strengthen the evidence supporting helmet therapy for infants with deformational plagiocephaly.

## Figures and Tables

**Table 1:** Subject information.

			No. of Patients (30)
Gender	Male		21
	Female		9
PMHx	Congenital muscular torticollis		17
	Sternocleidomastoid tumor		1
	Clavicle fracture		2
	DP with brachycephaly		2
	With physiotherapy sessions		22
Initial Age	Early	4–6 m	9
	Mid-age	7–9 m	13
	Late	10–12 m	8

DP: Deformational plagiocephaly; m: months.

**Table 2:** Primary Outcome (Paired T-test).

Primary Outcome			Pre-Helmet	%	Post-Helmet	%
CVAI (%)	Mild	0–5	4	13%	14	47%
	Moderate	6–10	23	77%	14	47%
	Severe	10+	3	10%	2	6%

**Table 3:** Change in Cranial Vault Asymmetry Index (CVAI) (%).

Change in CVAI *	N (%)
−3	1 (3.3)
−2	2 (6.7)
−1	3 (10.0)
0	7 (23.3)
1	2 (6.7)
2	5 (16.7)
3	3 (10.0)
4	5 (16.7)
5	0 (0.0)
6	1 (3.3)
7	1 (3.3)

* Change in CVAI = Initial CVAI − Last CVAI before wean off.

**Table 4:** Cross-tabulation between categorized percentages of initial CVAI and categorized last CVAI before wean off.

Initial CVAI	Last CVAI Before Wean Off			*p* Value
	Mild (0–5%)	Moderate (6–10%)	Severe (10+%)	
Mild (0–5%)	4 (100.0) (28.6)	0	0	0.044
Moderate (6–10%)	10 (43.5) (71.4)	12 (52.2) (85.7)	1 (4.3) (50.0)	
Severe (10+%)	0	2 (66.7) (14.3)	1 (33.3) (50.0)	

Bracket on the right side: row percentage; Bracket below: column percentage.

**Table 5:** Relationship between helmet compliance and change in CVAI after helmet therapy.

Helmet Compliance (Average Hours per Day)	CVAI		*p* Value
	Improved	No Change/Deteriorated	
0–5	2 (18.2) (16.7)	9 (81.8) (50.0)	0.049
6–11	5 (62.5) (41.7)	3 (37.5) (16.7)	
12–17	3 (33.3) (25.0)	6 (66.7) (33.3)	
≥18	2 (100.0) (16.7)	0 (0.0) (0.0)	

Bracket on the right side: row percentage; Bracket below: column percentage.

**Table 6:** Linear regression models on change in CVAI using different potential factors.

Model	Independent Variable	r^2^	SE	Coefficient (95% CI)	*p* Value
1	Age at recruitment	0.004	0.182	−0.04 (−0.437, 0.309)	NS
2	Sex (Ref: Male)	0.025			
	Female		0.968	−0.825 (−2.808, 1.157)	NS
3	Initial deformity level (Ref: N/A)	0.115			
	Clavicle fracture		1.869	−1.588 (−5.446, 2.270)	NS
	Brachycephaly		1.869	−2.088 (−5.946, 1.770)	NS
	Sternocleidomastoid tumor		2.573	0.412 (−4.898, 5.722)	NS
	Sternocleidomastoid tightness		2.573	2.412 (−2.898, 7.722)	NS
4	Compliance of helmet use (Ref: 0–5 h)	0.283			
	6–11		1.005	2.557 (0.492, 4.622)	0.017
	12–17		0.972	2.515 (0.518, 4.512)	0.016
	18+		1.102	3.182 (0.334, 6.598)	0.027
5	Wearing pattern (Ref: N/A)	0.365			
	Daytime		2.117	−0.167 (−4.518, 4.185)	NS
	Nighttime		2.144	3.000 (−1.407, 7.407)	NS
	Full time		2.157	2.375 (−2.059, 6.809)	NS

Dependent variables: change in CVAI (%); Dummy variables have been applied to sex, initial deformity level, compliance of helmet use, and wearing pattern.

**Table 7:** Stepwise linear regression models on change in CVAI using compliance of helmet use controlled by age at recruitment, sex, initial deformity, and wearing pattern.

Model	Compliance of Helmet Use (Ref: 0–5 h)	Age at Recruitment	Sex (Ref: Male)	Initial Deformity Level (Ref: N/A)	Wearing Pattern (Ref: N/A)	r^2^	SE	Coefficient (95% CI)	*p* Value
1		No controlling factor included							
						0.283			
	6–11						1.005	2.557 (0.492, 4.622)	0.017
	12–17						0.972	2.515 (0.518, 4.512)	0.016
	18+						1.102	3.182 (0.534, 6.598)	0.027
2		✓							
						0.285			
	6–11						1.048	2.494 (0.336, 4.653)	0.025
	12–17						1.012	2.574 (0.488, 4.659)	0.018
	18+						1.111	3.113 (0.510, 6.637)	0.031
3		✓	✓						
						0.299			
	6–11						1.066	2.416 (0.216, 4.615	0.033
	12–17						1.030	2.494 (0.368, 4.619)	0.023
	18+						1.133	3.189 (0.587, 6.765)	0.038
4		✓	✓	✓					
						0.387			
	6–11						1.245	2.282 (−0.323, 4.887)	NS
	12–17						1.220	2.655 (0.101, 5.209)	0.042
	18+						1.432	3.194 (0.046, 7.133)	0.048
5		✓	✓	✓	✓				
						0.409			
	6–11						1.520	−0.189 (−3.411, 3.033)	NS
	12–17						1.878	1.148 (−2.833, 5.128)	NS
	18+						2.064	1.819 (−3.616, 7.253)	NS

Dependent variables: change in CVAI (%); Dummy variables have been applied to sex, initial deformity level, compliance of helmet use, and wearing pattern. NS: Not statistically significant. ✓ indicates which factor(s) has been included in the “stepwise” linear regression models.

**Table 8:** Change in CVAI by helmet wearing pattern.

Wearing Pattern	Sample Size	Minimum	Minimum	Mean	SD
Daytime	13	−3	4	−1.5	1.819
Nighttime	9	−1	7	3.00	2.449
Fulltime	8	0	4	2.38	1.685

**Table 9:** Change in CVAI by initial age.

Initial Age (m)	Sample Size	Minimum	Maximum	Mean	SD
<7	9	−3	4	1.78	2.386
7–9	13	−2	6	1.15	2.375
>9	8	−1	7	1.63	2.774

**Table 10:** Outcome of 2 pairs of twins: Case 1a and Case 1b, as well as Case 2a and Case 2b.

Case No.	Start Age (m)	Initial CVAI (%)	Last CVAI (%)	Change in CVAI (%)	Initial ML—AP(mm)	Last ML—AP (mm)	Change in Cephalic Ratio
1a	10	6	5	1	N/A	N/A	N/A
1b *	8	4	4	0	131–123	139–136	1.07 => 1.02
2a *	11	7	7	0	146–138	152–150	1.06 => 1.02
2b	11	15	11	4	N/A	N/A	N/A

* Individuals who also had brachycephaly.

## Data Availability

The data that support the findings of this study are available from the corresponding author upon reasonable request.

## Abbreviations

The following abbreviations are used in this manuscript:

AP	Anteroposterior
CI	Confidence interval
CMT	Congenital muscular torticollis
CR	Cephalic ratio
CVAI	Cranial vault asymmetry index
DP	Deformational plagiocephaly
ML	Mediolateral
NS	Not statistically significant
PMHx	Past medical history
SE	Standard error
UCS	Unilateral coronal synostosis
